# Comparing the different morphotypes of a fish pathogen - implications for key virulence factors in *Flavobacterium columnare*

**DOI:** 10.1186/1471-2180-14-170

**Published:** 2014-06-26

**Authors:** Elina Laanto, Reetta K Penttinen, Jaana KH Bamford, Lotta-Riina Sundberg

**Affiliations:** 1 Centre of Excellence in Biological Interactions, Department of Biological and Environmental Science and Nanoscience Center, University of Jyväskylä, Jyväskylä, Finland

## Abstract

**Background:**

*Flavobacterium columnare* (Bacteroidetes) is the causative agent of columnaris disease in farmed freshwater fish around the world. The bacterium forms three colony morphotypes (Rhizoid, Rough and Soft), but the differences of the morphotypes are poorly known. We studied the virulence of the morphotypes produced by *F. columnare* strain B067 in rainbow trout (*Onconrhynchus mykiss*) and used high-resolution scanning electron microscopy to identify the fine structures of the cells grown in liquid and on agar. We also analysed the proteins secreted extracellularly and in membrane vesicles to identify possible virulence factors.

**Results:**

Only the Rhizoid morphotype was virulent in rainbow trout. Under electron microscopy, the cells of Rhizoid and Soft morphotypes were observed to display an organised structure within the colony, whereas in the Rough type this internal organisation was absent. Planktonic cells of the Rhizoid and Rough morphotypes produced large membrane vesicles that were not seen on the cells of the Soft morphotype. The vesicles were purified and analysed. Two proteins with predicted functions were identified, OmpA and SprF. Furthermore, the Rhizoid morphotype secreted a notable amount of a small, unidentified 13 kDa protein absent in the Rough and Soft morphotypes, indicating an association with bacterial virulence.

**Conclusions:**

Our results suggest three factors that are associated with the virulence of *F. columnare*: the coordinated organisation of cells, a secreted protein and outer membrane vesicles. The internal organisation of the cells within a colony may be associated with bacterial gliding motility, which has been suggested to be connected with virulence in *F. columnare*. The function of the secreted 13 kDa protein by the cells of the virulent morphotype cells remains unknown. The membrane vesicles might be connected with the adhesion of cells to the surfaces and could also carry potential virulence factors. Indeed, OmpA is a virulence factor in several bacterial pathogens, often linked with adhesion and invasion, and SprF is a protein connected with gliding motility and the protein secretion of flavobacteria.

## Background

Understanding the behaviour of pathogenic bacteria is a key component in elucidating host-pathogen interactions. The visualisation of the physical characteristics of bacteria, detailing the organisation and interactions between cells in different culture conditions, can provide new insights into the ecology of diseases and reveal why some bacteria are more difficult to eradicate than others. Indeed, bacterial cells often have structures that facilitate surface adhesion, biofilm formation and cell-cell interactions [1-3].

The ubiquitous ability of bacteria to form biofilms can influence virulence and promote persistent infections [4-6]. Bacteria in the biofilm are covered by an extracellular polymeric substance (EPS) layer that protects the cells from hostile environmental factors [7]. The EPS layer is comprised of a complex mixture of proteins, DNA and other materials, like outer membrane vesicles (OMVs). OMVs are abundant in the extracellular material of many gram-negative bacteria, including *Helicobacter pylori*, *Myxococcus xanthus* and *Pseudomonas aeruginosa*[8-10], and they are often associated with virulence. OMVs have also been detected in the fish pathogens *Flavobacterium psychrophilum* and *Flavobacterium columnare*[11,12]. A proteome analysis from the extracellular matrix proteins of *P. aeruginosa* PAO1 revealed that the OMVs constituted a large amount of the proteins in the biofilm [13]. The same study compared the proteomes of the OMVs from planktonic cells and cells in biofilm, which were observed to differ substantially. The planktonic OMVs of *P. aeruginosa* contained virulence factors such as LasA protease precursor, elastase LasB and alkaline protease whereas these were missing from the biofilm OMVs, indicating that planktonic cells may be important mediators of disease [13]. The role of OMVs has also been studied extensively in many other pathogenic bacteria, and there is no doubt of their significant role in the virulence of bacterial pathogens [14].

*F. columnare*, a member of Bacteroidetes, is a major bacterial pathogen of farmed freshwater fish around the world [15,16]. During the warm water period, the bacterium can be isolated from nature and fish tanks, both from biofilms and free water [17]. It is known that *F. columnare* can survive outside the fish host for long periods [18] and may respond to stressful conditions by entering into a viable but non-dividing state [12]. However, the infection mechanisms in this fish pathogen are still largely unknown.

We have previously observed that in the laboratory *F. columnare* can be induced to form different colony morphotypes by exposure to phage infection, starvation and serial culture [18,19]. Only the ancestral Rhizoid type has been shown to be virulent in fish, in which the derivative Rough and Soft types are non-virulent [18-20]. Therefore, identification of the structures and cell organisation of these virulent and non-virulent types can provide valuable information on how bacteria behave outside the host and offer clues about the possible virulence mechanisms. In this study we used high-resolution scanning electron microscopy (HR-SEM) to observe the cell organisation architecture and identify cell surface structures in both virulent (Rhizoid) and its derivative non-virulent (Rough and Soft) morphotypes of *F. columnare* strain B067 under different culture conditions. The parental Rhizoid type was originally isolated from a diseased trout (*Salmo trutta*), the Rough type was obtained by phage selection [19] and the Soft type appeared spontaneously during culture. Of the morphotypes, Rhizoid and Soft are able to form spreading colonies on agar [19], Sundberg et al., unpublished observations], which often indicates the ability for gliding motility, but may not always be in direct association [21]. As previous electron microscopy studies on *F. columnare* are scarce [12,22-25], information is needed on how bacterial cells with different levels of virulence interact with each other and with surfaces. Our aim is to discover the connections between bacterial cell characteristics and virulence.

## Results

### Virulence of the different colony morphotypes

**Figure 1:**
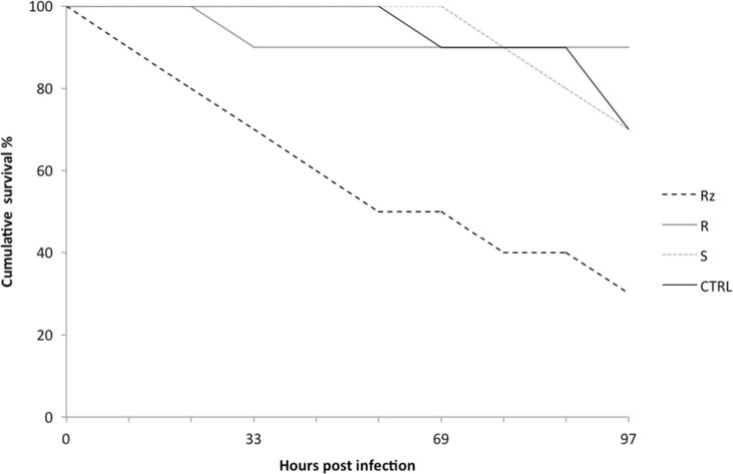
**Virulence of different morphotypes of *F. columnare.*** Rainbow trout (*Oncorhynchus mykiss*) survival after challenge with Rhizoid, Rough and Soft morphotypes of *F. columnare*.

Rainbow trout fry were exposed to Rhizoid, Rough and Soft morphotypes of *F. columnare* in a bath challenge, and the signs of disease and morbidity were recorded. Bacteria of the different colony types caused significant differences in fish survival (Kaplan-Meier survival analysis, χ^2^ = 12.007, df = 3, p = 0.007). The survival of the fish infected with the Rhizoid type was significantly lower than those infected with the Rough or Soft types (p-values, compared to Rhizoid, 0.008 and 0.036, respectively). The outcomes of the infections with Rough and Soft types were comparable to those of the negative control (p-value 0.032 for Rhizoid vs. negative control) (Figure 1).

### Surface structure of the colonies

**Figure 2:**
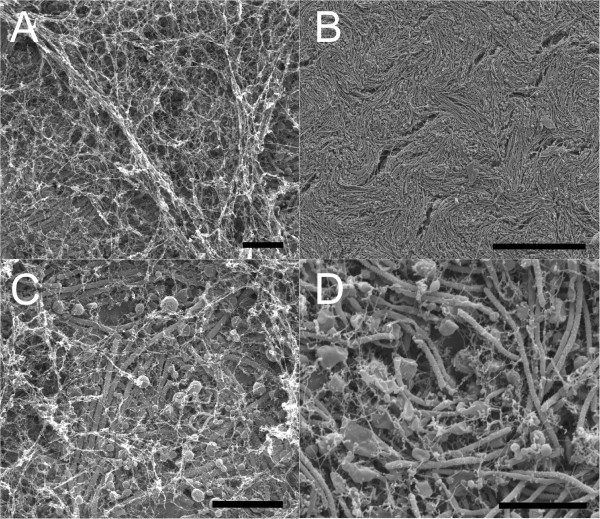
**Colony surfaces of the Rhizoid and Soft morphotypes of *F. columnare *B067.** The cells were grown on a filter paper on top of a Shieh agar plate and were visualised under HR-SEM. **Panel A**: Biofilm of the Rhizoid morphotype covered with an extracellular layer. **Panel C**: A closer view of a typical area where the surface layer is thin or missing, and large vesicles are visible (size approximately 1 μm). **Panels B and D**: Views of the colony/biofilm surface of the Soft morphotype where the fibrous layer found in other morphotypes was not detected. For the surface of the Rough morphotype, see Additional file 1. Notice the abundance of large vesicles in the Soft morphotype. Scale bar in A is 10 μm, in B, 40 μm, and in C and D, 4 μm.

To observe the surface structures of the bacterial colonies formed by the three different morphotypes, the colonies were grown on a filter paper and visualised under SEM. Biofilms of Rhizoid and Rough morphotypes were covered by a thick layer of extracellular filamentous material that was absent in the biofilm of the Soft morphotype (Figure 2 and Additional file 1). However, the layer covering the biofilm of the Rhizoid morphotype was not as complete as in the Rough morphotype, as cells were seen underneath (Figure 2A). In the Rhizoid and Soft morphotypes, the bacterial cells were accompanied by large vesicles with widely ranging sizes (up to 1.5 μm in diameter) (Figures 2C and D). Neither vesicles nor cells were seen underneath the thick extracellular material layer of the biofilm of the Rough morphotype (Additional file 1). Typical for the colony of the Soft morphotype were the wave-like arrangements formed by the cells with deep pores in regular intervals (Figure 2B).

### Internal structure of the colony types

**Figure 3:**
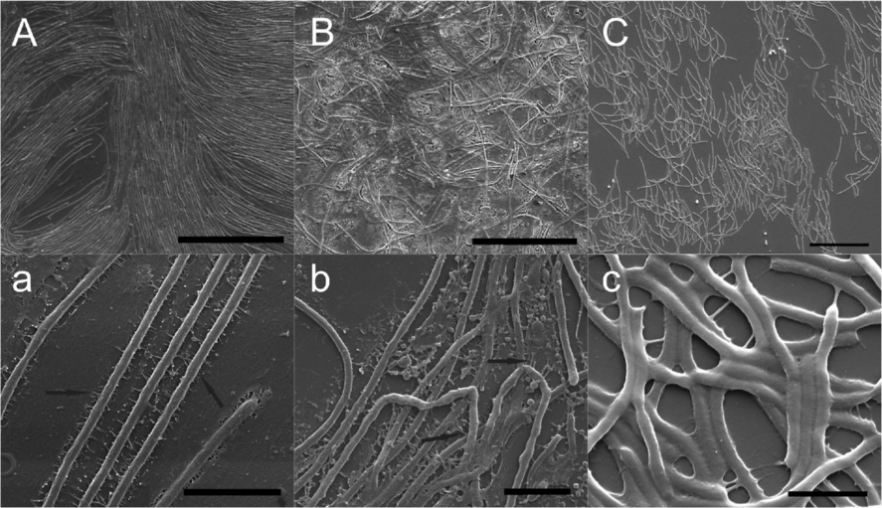
**Internal structure of colonies formed by Rhizoid, Rough and Soft morphotypes of *F. columnare *B067. ***F. columnare* cells were grown between a glass slide and Shieh agar and visualised under HR-SEM. **Panel A**: Rhizoid morphotype, **Panel B**: Rough morphotype and **Panel C**: Soft morphotype. A closer view of the cells can be seen underneath each panel marked with a matching lower case letter. Short filaments attaching cells to the surface and connecting cells are indicated by arrows in **3a** and **3b**. Scale bars in panels **A** to **C **20 μm and in **panels a to c** 2 μm.

**Figure 4:**
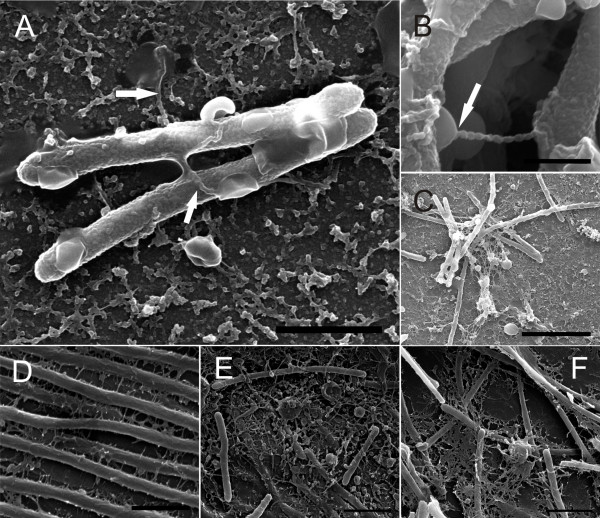
**Surface structures and vesicles observed in *F. columnare *B067 cultured in liquid and on agar. Panels A to C** represent the planktonic cells, and **panels D to F** show cells grown on agar. **Panel A**: A typical view of the planktonic cells of the Rhizoid morphotype. Vesicles and rope-like structures attaching vesicles to the cells (arrows) were abundant. **Panel B**: A closer view of the rope-like structure connecting a vesicle (arrow) and a cell from the Rough morphotype. **Panel C**: Typical clusters of cells with vesicles and long filaments in Rough morphotype. **Panel D**: An example of the short filaments on the cells of the Rhizoid morphotype. **Panels E and F**: cells and vesicles in a colony of the Rough morphotype. The scale bar in **Panel A** was 1 μm, in **B**, 500 nm, in **C**, 4 μm, in **D**, 1 μm, and in **Panels E and F**, 2 μm.

The cell organisation and internal structure of the colonies of the different morphotypes grown between a glass slide and a Shieh agar plate were visualised under SEM (Figure 3 and Figures 4D-F). Cells of the virulent Rhizoid morphotype formed organised structures on the glass slide, with characteristics of coordinated social movement (Figure 3A). The bacteria were attached to the surface and to each other by numerous thin fimbriae-like strings (Figures 3a and 4D). Cells in the colony of the non-virulent Rough morphotype were randomly scattered on the glass surface without any organised population structure, in contrast to that observed in the virulent Rhizoid type (Figure 3B). Cells of the Rough morphotype also exhibited slightly thicker fimbriae than the Rhizoid type that did not appear regularly on the cell surfaces (Figures 3a and b). Membrane vesicles were observed on the surface of both Rhizoid and Rough morphotypes (Figures 3a and b). Numerous vesicles of different sizes were detached from and scattered around the cells. Smaller vesicles (approximately 200 nm) formed vesicle chains, middle size vesicles (approximately 500 nm to 1 μm in size) were also abundant and a few larger vesicles (approximately 1,5 μm) were seen (Figures 4E and F). Furthermore, the shape of the Rough type cells was uneven compared to Rhizoid type cells. The non-virulent Soft morphotype cells formed wave-like ‘dunes’ on the glass (Figure 3C). Fimbriae connecting the bacteria to the glass were observed, and the cells appeared to be attached to the surface more along their length, but the vesicles were absent from the cells of the Soft morphotype (Figure 3c).

### Planktonic cells of the colony types

**Figure 5:**
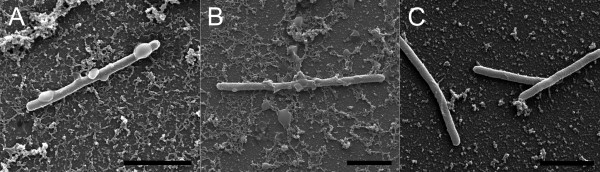
**Planktonic cells of Rhizoid, Rough and Soft morphotypes of *F. columnare *B067.** A typical view of the cells grown in liquid medium visualised under HR-SEM of the three morphotypes. Large vesicles were typical on the surface and surroundings of the **A)** Rhizoid and **B)** Rough morphotypes. **Panel C**: On the surface of the cells of the Soft morphotype, only small blebs were seen. The scale bar was 2 μm.

**Figure 6:**
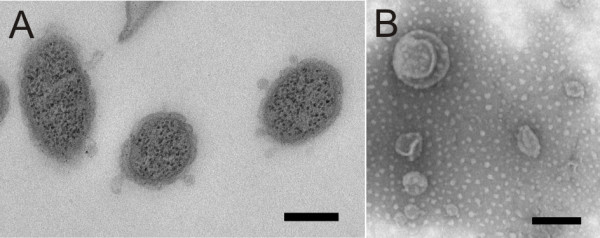
**OMVs of Rhizoid morphotype *of F. columnare *B067. Panel A**: Outer membrane vesicles visualised under TEM from the thin sections of the cells of the *F. columnare*; and **Panel B**: Purified vesicles under TEM. In the thin sections, the size of the observed vesicles was under 100 nm, whereas the purified vesicles ranged from 60 to 350 nm. The scale bar was 200 nm.

Liquid bacterial cultures were visualised on Concanavalin A (ConA) plates under SEM. Large surface-associated vesicles were seen on cells of the Rhizoid and Rough morphotypes, but not on those of the Soft type (Figures 5, 4A and C). Individual cells had several vesicles that were spread evenly across the cell length. The surface of the vesicles was smoother than the surface of the bacterial cell, indicating that the membrane of the vesicles may be lipid, which was confirmed by transmission electron microscopy (TEM) analysis (see later). Also, in the Rhizoid and Rough morphotypes the bacteria produced thick rope or pearl chain -like structures to attach to each other and to the surface (Figures 4A and B). The liquid cultures were observed to contain aggregates (Figure 5 and Additional file 2) that were designated to originate from the growth medium according to the control sample containing only Shieh medium (Additional file 3C). A wider view of the typical samples of planktonic cells visualised under SEM is provided in Additional file 2. TEM analysis did not reveal a difference between the OMVs in the Rhizoid and Rough morphotypes in liquid culture, which is consistent with the results received by SEM. Vesicles with a bilayer and clustered electron-dense material were seen on the surface of both morphotypes, but their size was less than in the SEM analysis (average 50 nm under TEM vs. 100–500 nm under SEM) (Figure 6A).

### Differences in the extracellularly secreted protein profiles by the colony types

**Figure 7:**
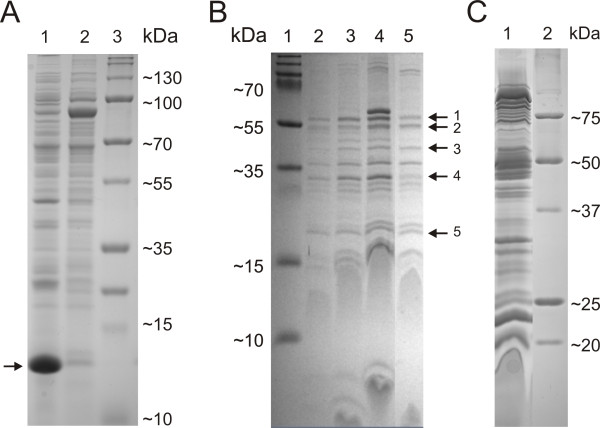
**Protein profiles of *F. columnare *B067 morphotypes. Panel A**: Extracellular protein profiles of the Rhizoid (Lane 1) and Rough (Lane 2) morphotype of the *F. columnare* strain B067 run in a 14% Tricine SDS-PAGE -gel. The protein band that was analysed further by nanoLC-ESI-MS/MS is indicated by an arrow. PageRuler Plus Prestained Protein Ladder (Thermo Scientific) MW marker was used for Lane 3. **Panel B**: OMV fractions from the 20–45% OptiPrep gradient run in a 14% Tricine SDS-PAGE gel. OMVs were purified from both Rhizoid and Rough morphotypes. Lane 1: PageRuler Plus Prestained Protein Ladder (Thermo Scientific) MW marker. Lanes 2 to 4: Rhizoid morphotype, three light-scattering fractions. Lane 5: Rough morphotype, middle fraction. Arrows indicate the protein bands that were further analysed from the middle fraction of the Rhizoid morphotype by nanoLC-ESI-MS/MS. **Panel C**: Outer membrane protein profile of the Rhizoid morphotype (Lane 1) and Precision Plus Protein™ Standard (BioRad) MW marker (Lane 2) run in a 16% SDS-PAGE.

The proteins concentrated from the supernatant of 18-hour cultures of the three morphotypes were analysed on Tricine-SDS-PAGE (Figure 7A). A notable amount of a small protein was present in the profile of the Rhizoid morphotype that was found missing or in very low amounts in the Rough and Soft morphotypes (MW approximately 13 kDa) (data for the Soft morphotype not shown). The protein was identified using nanoLC-ESI-MS/MS as a hypothetical protein FCOL_04265 in *F. columnare* ATCC 49512 (Table 1), but the function of the protein is unknown. Furthermore, this protein is specific for *F. columnare* and is not present in its close relatives, *F. psychrophilum* and *F. johnsoniae*.

**Table 1:** Identified proteins

**Band name**	**Size (kDa) on gel**	**Protein identification by nanoLC-ESI-MS/MS**	**Match to ORF [Accession number in NCBI]**	**Best hit in BLAST: identity % (query cover %)**	**Predicted size (kDa)**
1	≈ 55	Hypothetical protein	FCOL_07765 [YP_004942163.1]	FCOL_11410 47% (100%)	53.5
2	≈ 55	OmpA family outer membrane protein P60	FCOL_09105 [YP_004942423.1]	FP0156 Outer membrane protein precursor; OmpA family P60 [*F. psychrophilum* JIP02/86] 70% (100%)	50.2
3	45	Hypothetical protein	FCOL_02860 [YP_004941210.1]	FP1486 Protein of unknown function [*F. psychrophilum* JIP02/86] 55% (98%)	44.5
4	35	Hypothetical protein	FCOL_08865 [YP_004942378.1]	FP0017 Putative cell surface protein precursor SprF [*F. psychrophilum* JIP02/86] 68% (94%)	37.9
5	20	Hypothetical protein	FCOL_11765 [YP_004942947.1]	FP2260 Protein of unknown function precursor [*F. psychrophilum* JIP02/86] 48% (100%)	24.4
ECP	13	Hypothetical protein	FCOL_04265 [YP_004941480.1]	-	18,1

Protein identification of the membrane vesicle contents and the extracellular protein of *Flavobacterium columnare* B067 by nanoLC-ESI-MS/MS, and the subsequent identification by BLAST search. ORF annotation refers to *F. columnare* ATCC 49512 complete genome [NCBI: NC_016510].

### Characterisation of OMVs

The OMVs of the Rhizoid morphotype were isolated and purified. The purification was done by density gradient centrifugation and resulted in three light-scattering bands. The bands were pelleted and visualised under TEM, which revealed the different sizes of the purified vesicles, ranging from approximately 60 nm to 350 nm (Figure 6B). These vesicles were run on a 14% Tricine SDS-PAGE (Figure 7B) and compared to the outer membrane protein profile of the Rhizoid morphotype without any notable additional bands (Figure 7C). Five protein bands from vesicle profile were commercially analysed in more detail by nanoLC-ESI-MS/MS. One protein band was identified as the OmpA outer membrane P60 of the *F. columnare* strain ATCC 49512 (Table 1). Others were identified as hypothetical proteins of the same bacterium. The resulting proteins were compared to database sequences using the BLAST algorithm and according to their match to the *F. columnare* ATCC 49512 genome. One of the identified proteins with an unknown function recorded a hit for flavobacterial gliding motility protein SprF of *F. psychrophilum* and *F. johnsoniae,* and based on amino acid similarity, it was designated as SprF (Table 1).

## Discussion

Genetic properties and whole cell protein profiles of the different colony morphologies of *F. columnare* have been studied previously, but no differences have been detected [18]. We used HR-SEM to study parental virulent (Rhizoid) and its derivative non-virulent (Rough and Soft) morphotypes of the same bacterial strain in different culture conditions, and also compared their extracellular protein profiles. We found clear differences in the cell organisation, cell surface structures and extracellular protein profiles between the virulent and non-virulent morphotypes and suggested new factors that are potentially connected to the virulence of *F. columnare.* Virulence of the Rhizoid type was clearly high in rainbow trout fry, whereas the Rough and Soft types produced mortality rates comparable to the control treatment (Figure 1). The virulent Rhizoid type secreted a high amount of a small (approximately 13 kDa) protein, whose function is unclear, but which is not found in other bacterial species. Furthermore, our experiments revealed OMVs with variable sizes in the Rhizoid and Rough morphotypes. The vesicles were found to contain proteins with unknown functions and a OmpA-family protein, which is associated with virulence in other bacterial pathogens (see later).

Biofilms are important reservoirs of bacteria in nature [26]. Therefore, it is important to understand how bacteria form and interact within biofilms. We visualised both the surface and internal structures of the bacterial biofilm of different colony types of *F. columnare* grown on agar. We found that the virulent Rhizoid morphotype produced an organised biofilm within the colony with indications of social movement, whereas in the phage-resistant Rough morphotype this behaviour was absent, and the cells were randomly scattered (Figure 3). Also, according to the surface view, the Soft type colony had an organised structure (Figure 2). This was not clear when the internal structure of the colony was studied, which is probably due to the fact that cells of the Soft type are not adherent and are therefore unable to stay fully attached to the visualised glass slide. As both Rhizoid and Soft types can form spreading colonies on agar, it is possible that the organisation of cells within the colony is associated with gliding motility. When the surface of the colonies was studied, the Rhizoid and Rough morphotype cells were observed to be covered by a fibrous extracellular layer that was missing in the non-virulent Soft morphotype (Figure 2). The fibrous surface layer may protect the bacteria from environmental stressors, such as protozoan grazing [4], but it may also be connected to the strong adherence of the colonies on agar. The Soft type, missing this fibrous layer, is indeed non-adherent, compared to the Rhizoid and Rough types [20].

Extracellularly secreted proteins have been suggested to be important for virulence in *F. columnare*[27,28]. In the current study, the comparison of extracellular protein profiles revealed a major difference between the virulent and non-virulent morphotypes. A notable amount of a small protein (MW ~ 13 kDa) was present in the protein profile of the Rhizoid type that was absent or present only in small quantities in the Rough and Soft types. The protein was designated as a hypothetical protein of *F. columnare*, but no function for the protein was identified. We have also observed this protein in the Rhizoid morphotypes of two other virulent *F. columnare* strains (unpublished), and in minor quantities in the non-virulent Rough morphotypes of these strains. Due to its association with the Rhizoid colony type, we suggest that it could have a role in the virulence of *F. columnare*. However, the exact function of this protein requires future elucidation.

In previous studies on *F. columnare*, evidence has been found for narrow extensions and slender projections from the outer membranes of the cells [22-24]. Furthermore, small membrane vesicles and extracellular polysaccharide substances were observed in recent studies [12,25], but their role has not been confirmed, although it has been observed that *F. columnare* can rapidly adhere to and colonise surfaces and initiate biofilm formation [25]. OMVs are described in a majority of gram-negative bacteria, and they play a significant role in the virulence of bacteria [14]. Vesicles can contain toxins or adhesins that are delivered directly into the host cells [14,29-31]. Moreover, OMVs are a functional part of natural biofilms, having proteolytic activity and binding antibiotics, such as gentamycin [10]. Generally, the size of an OMV ranges between 50 and 250 nm [29]. We observed two kinds of membrane vesicles in *F. columnare* grown in liquid. Under SEM, large (100–500 nm) vesicles were abundant on the surface of the Rhizoid and Rough (but not Soft) bacteria, as well as smaller (approximately less than 100 nm) surface vesicles, which also formed chain-like structures between individual bacterial cells. When thin-sectioned cells were visualised under TEM, the vesicles were observed to have a lipid bilayer, but the size was approximately 50 nm. TEM analysis of the purified vesicles revealed vesicles ranging in size from 60 to 350 nm. The reason for the absence of the large vesicles in the thin-sectioned samples is unclear, but it could be due to the sampling process. SEM analysis suggests that the large vesicles may be connected to the surface adhesion of the bacteria. The bacteria have several vesicles on their surface, which seem to erupt by contact, anchoring the bacteria to the surrounding surface. This result was supported by an analysis of vesicle contents, where the OmpA family outer membrane protein was identified. OmpA is often associated with adhesion to host tissues [32]. Indeed, the Rhizoid and Rough morphotypes are highly adherent, whereas the Soft morphotype (lacking the vesicles in liquid culture) is not [18]. However, the Soft type also produced large vesicles when grown on agar, though it is not clear whether these vesicles are the same as those found in liquid cultures or in the Rhizoid and Rough types.

Although the function of the small vesicles and pearl-like vesicle chains observed in *F. columnare* was not analysed in depth in the current study, in TEM analysis they were shown to contain electron-dense material. The vesicle chains in the liquid cultures typically connected the cells to each other and to their surroundings. Usually there was a larger vesicle at the end of the chain, which in some cases appeared to have erupted by contact, possibly serving as an adhesin. Similar to *F. columnare*, small vesicles and their chain-like formations have been found in *F. psychrophilum. F. psychrophilum* produces small vesicles that bleb from the surface in pearl-like chain structures and exhibit proteolytic activity [11,33]. Although observed under both TEM and SEM, the nature of these pearl-like structures or ropes produced by all morphotypes of *F. columnare—*and whether they are ultrastructural artefacts caused by sample preparation—remains unclear. Recently, however, vesicle chains were also reported in *M. xanthus*, and were suggested to connect the cells in biofilms at the level of the periplasmic space, enabling the transfer of membrane proteins and other molecules between cells [34]. In contrast to *M. xanthus*, which had an increased abundance of vesicle chains in the biofilms, the vesicle chain-like structures observed in *F. columnare* were more common in the liquid cultures, though they were also observed in colonies (Figures 3 and 4).

In the initial protein identification, the proteins extracted from the vesicles remained hypothetical, except for one band, which was identified as the OmpA-family outer membrane protein P60 (see Table 1), but they all matched the *F. columnare* ATCC 49512 genome. After a basic local alignment search tool (BLAST) analysis, one protein was further identified as SprF. OmpA-family proteins are known to be virulence factors in several bacterial pathogens. The way in which OmpA-family proteins associate with *F. columnare* virulence is unclear, but our data implies that OmpA is involved with adhesion, and therefore might be a candidate virulence factor. Although the same protein band was present in the vesicles isolated from the non-virulent Rough type (Figure 7B), the virulence of the Rough type is probably affected by the loss of gliding motility. In addition, vesicles were not detected from the cells of the Soft morphotype that possess gliding motility, according to the spreading of colonies. Indeed, OmpA has been demonstrated to act as an adhesin and invasin, for example in *Pasteurella multocida*[35], several *E. coli* strains [36], *Neisseria gonorrhea*[32], *Leptospira interrogans* (causative agent of leptospirosis) [37], *Riemerella anatipestifer* (pathogen of domestic ducks) [38] and many other pathogens [39]. The protein has a strong immunogenic capacity [36,40]. In *F. psychrophilum*, OmpA has been identified as a promising candidate for the immunisation of rainbow trout against bacterial cold-water disease [41]. The role of the *F. columnare* OmpA-family protein for adhesion and invasion, and, on the other hand, as an immunogenic protein requires further study to reveal the mechanisms of how it interacts with the host tissue. However, the absence of functional genetic techniques hampers the genetic manipulation and verification of the role of OmpA as a virulence factor of *F. columnare*.

The protein identified as SprF is involved with flavobacterial gliding motility. In *F. johnsoniae*, Spr proteins (SprB together with SprC, SprD and SprF) are needed for the formation of spreading colonies on agar [21]. In *F. columnare*, the Rhizoid colony morphology (and corresponding gliding motility) is needed for virulence [18-20], possibly because of the role of flavobacterial gliding motility machinery as a type IX secretion system of virulence factors [42]. Indeed, SprF is needed for the secretion of SprB on the cell surface [21], but so far the specific role of SprF in *F. columnare* remains cryptic. Moreover, in the Rhizoid type, we observed numerous cell surface filaments that seemed to be situated at regular intervals along the cell, and appeared to attach bacterial cells to the glass surface and to neighbouring bacterial cells (Figure 3A). As these regularly appearing filaments were detected in lower numbers and in a less organised manner in the non-motile rough type, it is possible that these filaments are connected with gliding motility. It should be noted that the non-spreading Rough type colonies might not directly correlate to a loss of gliding motility. In *F. johnsoniae*, it has been observed that non-spreading colonies may not directly indicate loss of gliding motility, as this loss depends on whether mutations occur in *gld* or *spr* genes [21]. The surface adhesin SprB needed for flavobacterial gliding motility is a filament, approximately 150 nm long, on the cell surface [43,44]. As the structure of individual SprB proteins is fragile, and as the platinum sputter used in coating the samples can cover the finest structures, it is likely that the filaments visible in the Rhizoid type are adhesive structures other than SprB.

## Conclusions

Our results suggest candidate virulence factors for *F. columnare,* factors that are still poorly understood, despite the problems caused by columnaris disease in the aquacultural industry. Additional questions are raised, especially on the role of OmpA and other unidentified proteins carried within the vesicles and secreted outside the cell, on adhesion to surfaces and invasion into the fish host. Also, the loss of an organised internal structure within the colony in the phage-resistant Rough type bacteria suggests that connections between neighbouring cells and social behaviour might be important for virulence in *F. columnare*.

## Methods

### Bacterial cultures

*Flavobacterium columnare* strain B067 was originally isolated from diseased trout (*Salmo trutta*) in 2007, and was stored frozen at −80°C in Shieh medium [45] with glycerol (10%) and foetal calf serum (10%). The derivative Rough phenotype of the strain was obtained by exposure to phage FCL-1 (see [19] for details). The Soft morphotype was isolated as a spontaneous transformant from the Rhizoid type. Bacteria were grown in a Shieh medium at 24°C under a constant agitation of 110 rpm in an orbital shaker.

### Virulence experiments

The virulence of the bacteria producing the Rhizoid, Rough and Soft colony types of strain B067 was studied in an infection experiment using rainbow trout fry (*Onconrhynchus mykiss*, mean weight 0.57 g). Ten fish per colony type were individually exposed to 1×10^5^ colony-forming units of bacteria ml^−1^ in 100 ml of ground water for 1 hour (T = 25°C). As a control treatment, 10 fish were individually exposed to sterile growth medium. After exposure, the fish were transferred to a 1-litre aquaria with 500 ml of fresh ground water (T = 25°C), and disease signs and fish morbidity were monitored in two-hour intervals for 97 hours. Morbid fish that had lost their natural swimming buoyancy, and which did not respond to external stimuli, were considered dead, removed from the experiment and euthanatised by cutting the spinal cord to avoid the suffering of the fish. The experiment was conducted according to the Finnish Act on the Use of Animals for Experimental Purposes, under permission granted by the National Animal Experiment Board at the Regional State Administrative Agency for Southern Finland for L-RS. The virulence of the different colony types on rainbow trout infection was analysed by Kaplan-Meier survival analysis using IBM SPSS Statistics 20.

### Treatment of SEM samples

*F. columnare* cell and biofilm structures were studied in three replicates in three different culture conditions: in liquid, in biofilm grown on a filter and in biofilm formed between a glass slide and an agar plate. To visualise the structure of the cells in liquid culture, *F. columnare* culture was placed on a ConA plate, incubated for 30 minutes and fixed (see later). To study the organisation of cells in a colony, the bacteria were cultured on Shieh plates with a glass slide (18×18 mm) placed on top of the culture. After 48 hours, the slide was detached, fixed and processed for SEM. To study the colony structure on an agar plate, a sterile 0.45 μm cellulose nitrate filter was placed on the Shieh agar, and 50 μl of the bacteria was spread on top of it. After 24 hours of growth, the filter was detached, fixed and processed. As control samples for SEM visualisation, sterile Shieh medium, supernatant from the cultures and *Escherichia coli* and *Salmonella enterica* cells (both grown in Shieh medium) were used. Samples were fixed (2% glutaraldehyde in 0.1 M NaCacodylate buffer, pH7.4) and washed twice with 0.1 M NaCac buffer, osmicated at RT, and washed twice with 0.1 M NaCac buffer. The cells were then dehydrated by exposure to a graded series of ethanol washes [50%, 70%, 96% and 100% (2×); each 3 min]. Filter samples were dried using the critical point method. The samples were then coated with platinum using platinum splutter and observed with an FEI Quanta™ 250 FEG-SEM.

### Treatment of cells for TEM

Thin sections from the liquid cultures of Rhizoid and Rough morphotypes were also visualised using TEM. Samples were prepared from 5 ml of cultures grown for 16 hours, mixed with a final concentration of 3% glutaraldehyde and kept on ice for 45 minutes. The cells were washed three times with 0.1 M sodium phosphate buffer (pH 7.2). The pellet was overlaid with 1 ml of 1% osmium tetroxide in a phosphate buffer, washed once with the same buffer and then dehydrated in a rinsing ethanol series. The cells were embedded with Epon (Fisher) and sectioned.

### Extraction of proteins from the ECPs

Rhizoid, Rough and Soft morphotype cells of *F. columnare* were grown in 50 ml of Shieh medium for 18 hours and pelleted (Megafuge 1.0R, 2500 × *g*, 15 min). Supernatant was filtered (0.8/0.2 μm pore size, Supor® membrane, PALL Life Sciences). Proteins were concentrated from 50 ml of the filtered supernatant with Amicon® Ultra Centrifugal filters (Ultracel®, 10 K, Millipore) to 500 μl in final volume. The protein concentration was determined with the Bradford protein assay [46]. Samples were run in a 14% Tricine SDS-PAGE [47] at 80 V, 30 mA for 20 hours. One protein band was excised and further analysed.

### Isolation and analysis of membrane vesicles

The OMVs of the cells of the Rhizoid and Rough morphotypes of *F. columnare* strain B067 were isolated following the general outline for purification of natural OMVs in [48]. Bacteria were grown in 125 ml Shieh medium for 22–24 hours at RT with 110 rpm agitation. The cells were then removed by centrifugation (Sorvall RC-5, GSA- rotor, 10 400 × g, 30 min, RT) and the supernatant was filtered through a bottle-top filter unit (0.45 μm pore size, PES membrane, Nalgene), passing only vesicles less than 450 nm in size to the filtrate. The filtered supernatant was pelleted (Beckman coulter L-90 K, 45 Ti rotor, 60 000 × g, 2 h 30 min, 4°C) and resuspended in 1XPBS (phosphate buffer saline**)**. The pellet was loaded on top of a 20–45% OptiPrep gradient and centrifuged (Beckman Coulter L-90 K, SW 41 rotor, 49 000 × g, 17 h, 15°C). The light scattering fractions were collected, pelleted (Beckman coulter L-90 K, 70.1 Ti rotor, 54 000 × g, 3 h, 4°C) and resuspended in 1XPBS. The fractions were analysed under TEM. Samples were spotted on carbon-stabilised formvar-coated grids and fixed with 2% glutaraldehyde/0.1 M NaPOH for 1 minute and were washed three times with distilled H_2_O and stained with 1% phosphotungstate, pH 6.5 for 1 minute. Imaging was performed with a Jeol JEM-1400. Fractions were also run in a 14% Tricine SDS-PAGE [47] at 80 V, 30 mA for 22 hours. Five protein bands were excised and further analysed.

### Extraction of outer membrane proteins

The cell pellet from the ECP extraction of the Rhizoid morphotype was subjected to OMP extraction. The cells were disrupted by freeze-thawing three times, and cell debris was removed by centrifugation (5000 × g, 15 min, 4°C). Supernatant was then centrifuged (50 000 × g, 60 min, 4°C) and the pellet was suspended in 30 ml of 0.5% N-lauroylsarcosine/20 mM Tris–HCl, pH 7.2, and incubated for 20 minutes on ice in a cold room. Centrifugation was repeated, and the pellet was suspended in 4 ml of 0.5% N-lauroylsarcosine/20 mM Tris–HCl, pH 7.2, and centrifuged. The pellet was washed twice with 4 ml and suspended in 100 μl of 20 mM Tris–HCl. The sample was run in a 16% SDS-PAGE [49] at 100 V, 30 mA for 22 hours.

### Protein identification

Protein identification using nanoLC-ESI-MS/MS was performed by ProteomeFactory (Proteome Factory AG, Berlin, Germany). The MS system consisted of an Agilent 1100 nanoLCsystem (Agilent, Waldbronn, Germany), a PicoTip electrospray emitter (New Objective, Woburn, MA) and an Orbitrap XL or a LTQ-FT Ultra mass spectrometer (ThermoFisher, Bremen, Germany). Protein spots were in-gel digested by trypsin (Promega, Mannheim, Germany) and applied to nanoLC-ESI-MS/MS. Peptides were trapped and desalted on the enrichment column (Zorbax SB C18, 0.3 × 5 mm, Agilent) for five minutes using 2.5% acetonitrile/0.5% formic acid as an eluent, then they were separated on a Zorbax 300 SB C18, 75 μm × 150 mm column (Agilent) using an acetonitrile/0.1% formic acid gradient from 5% to 35% acetonitril within 40 minutes. MS/MS spectra were recorded data-dependently by the mass spectrometer according to the manufacturer’s recommendations. Proteins were identified using an MS/MS ion search with the Mascot search engine (Matrix Science, London, England) and the nr protein database (National Centre for Biotechnology Information, Bethesda, USA). The ion charge in the search parameters for ions from ESI-MS/MS data acquisition were set to ‘1+, 2+ or 3+’, according to the instrument’s and method’s common charge state distribution. The resulting proteins were compared to database sequences using the BLAST algorithm [50].

## Competing interests

The authors declare that no competing interests exist.

## Authors’ contributions

EL performed the experiments, participated in the sample preparation and design of the study and drafted the manuscript. RKP participated in the sample preparation and performed the virulence experiments and sequence comparisons. JKHB participated in the design of the study and helped to draft the manuscript. LRS conceived of the study, participated in its design and coordination and drafted the manuscript. All authors read and approved the final manuscript.

## Supplementary Material

Additional file 1 **A view of the colony surface of the Rough morphotype of *F. columnare.*** Only the extracellular material was seen on the colony surface of the Rough morphotype, and cells were not observed. The scale bar was 4 μm. Click here for file

Additional file 2 **A wider view of a typical sample of the planktonic cells from the three morphotypes visualised under HR-SEM.** Panel A: Rhizoid morphotype cells. Panel B: Rough morphotype cells. Panel C: Soft morphotype cells. The scale bar in A was 30 μm and in B and C, 40 μm. Click here for file

Additional file 3 **Controls used in the HR-SEM studies.** Panel A: *E. coli* cells grown in Shieh medium. Panel B: *E. coli* cells grown on a filter paper (on Shieh agar). Panel C: Sterile Shieh medium. No vesicles, vesicle chains or filaments were seen in the controls. The scale bar in Panel A was 5 μm and was 10 μm in Panels B and C. Click here for file
