# Supplementary material for: Comparing the different morphotypes of a fish pathogen - implications for key virulence factors in Flavobacterium columnare
Source: BMC Microbiol. 2014 Jun 26;14:170. doi: 10.1186/1471-2180-14-170 (PMC4094633; doi:10.1186/1471-2180-14-170)
Supplement: Additional file 1 — A view of the colony surface of the Rough morphotype of F. columnare. Only the extracellular material was seen on the colony surface of the Rough morphotype, and cells were not observed. The scale bar was 4 μm. [file 1471-2180-14-170-S1.pdf]

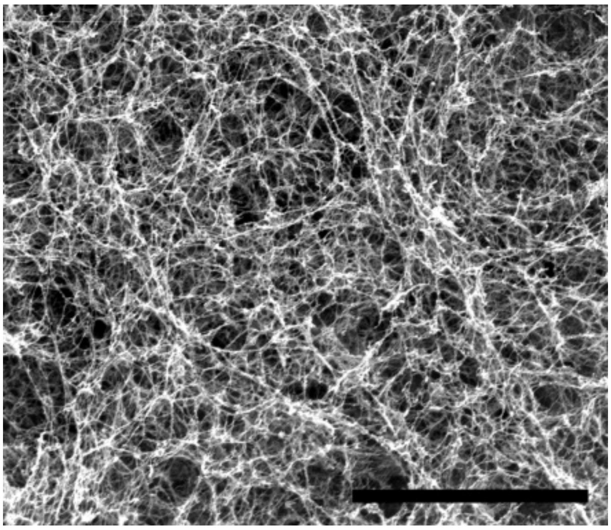

Additional file 1 (.pdf)
A view of the colony surface of the Rough morphotype of *F. columnare*
Only the extracellular material was seen on the colony surface of the Rough morphotype, and cells were not observed. The scale bar was 4 µm.
