# Supplementary material for: Comparing the different morphotypes of a fish pathogen - implications for key virulence factors in Flavobacterium columnare
Source: BMC Microbiol. 2014 Jun 26;14:170. doi: 10.1186/1471-2180-14-170 (PMC4094633; doi:10.1186/1471-2180-14-170)
Supplement: Additional file 2 — A wider view of a typical sample of the planktonic cells from the three morphotypes visualised under HR-SEM. Panel A: Rhizoid morphotype cells. Panel B: Rough morphotype cells. Panel C: Soft morphotype cells. The scale bar in A was 30 μm and in B and C, 40 μm. [file 1471-2180-14-170-S2.pdf]

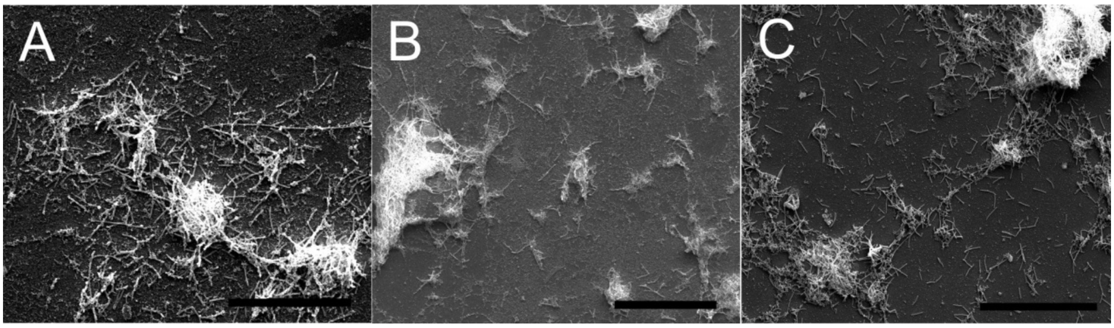

Additional file 2 (.pdf)
A wider view of a typical sample of the planktonic cells from the three morphotypes visualised under HR-SEM
Panel A: Rhizoid morphotype cells. Panel B: Rough morphotype cells. Panel C: Soft morphotype cells. The scale bar in A was 30 µm and in B and C, 40 µm.
