# Supplementary material for: Comparing the different morphotypes of a fish pathogen - implications for key virulence factors in Flavobacterium columnare
Source: BMC Microbiol. 2014 Jun 26;14:170. doi: 10.1186/1471-2180-14-170 (PMC4094633; doi:10.1186/1471-2180-14-170)
Supplement: Additional file 3 — Controls used in the HR-SEM studies. Panel A: E. coli cells grown in Shieh medium. Panel B: E. coli cells grown on a filter paper (on Shieh agar). Panel C: Sterile Shieh medium. No vesicles, vesicle chains or filaments were seen in the controls. The scale bar in Panel A was 5 μm and was 10 μm in Panels B and C. [file 1471-2180-14-170-S3.pdf]

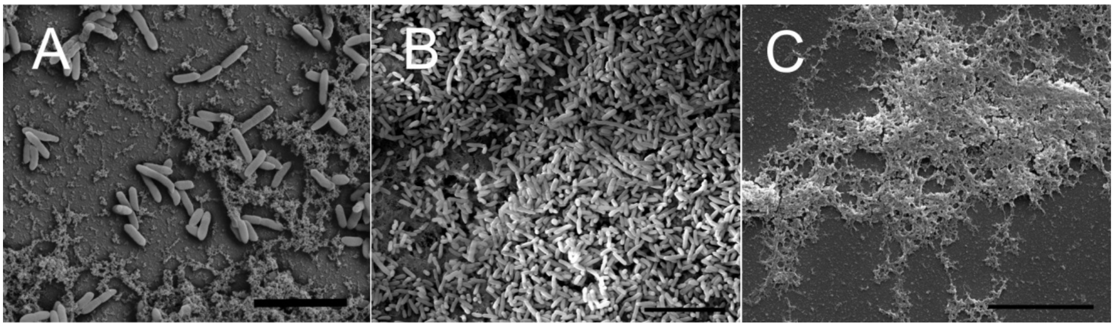

Additional file 3 (.pdf)
Controls used in the HR-SEM studies
Panel A: *E. coli* cells grown in Shieh medium. Panel B: *E. coli* cells grown on a filter paper (on Shieh agar). Panel C: Sterile Shieh medium. No vesicles, vesicle chains or filaments were seen in the controls. The scale bar in Panel A was 5 µm and was 10 µm in Panels B and C.
